# Measuring surprisal in sound sequences

**DOI:** 10.3758/s13428-026-03153-3

**Published:** 2026-08-24

**Authors:** Andrey Anikin

**Affiliations:** Division of Cognitive Science, Department of Philosophy, Lund University, Box 192, SE-221 00 Lund, Sweden

**Keywords:** Auditory attention, Salience, Shannon surprisal, Bayesian surprise, Self-similarity

## Abstract

Sensory input that violates prior expectations attracts attention, making unpredictability an important perceptual property to measure. In the auditory modality, knowing what sounds will be perceived as surprising, and therefore salient, is relevant both for studying vocal communication and for applied purposes such as managing noise pollution. Focusing on sequences of animal vocalizations and environmental sounds as ecologically important acoustic stimuli, I describe and benchmark several algorithms for measuring their perceived unpredictability. Information-theoretical approaches include Shannon surprisal and Bayesian surprise, both implemented here to detect deviant stimuli based on distributional acoustic properties. The second group of algorithms is based on detecting spectro-temporal recurrence assessed with autocorrelation functions (ACF surprisal) and self-similarity matrices (SSM novelty). The third approach uses neural networks. Based on the ratings of the predictability of 300 synthetic acoustic sequences by 195 human listeners, Shannon surprisal and SSM novelty capture the perceived unpredictability that is due to spectral variability, whereas ACF surprisal taps into the perceptual impact of irregular rhythm. Most algorithms converge on the time scale of about 1 s as the most perceptually relevant for spectral variability, which is consistent with the hypothesis that the perception of unpredictability stems from a relatively limited amount of auditory input held in short-term memory. Together, the presented open-source algorithms offer powerful and flexible tools for measuring acoustic surprisal and studying auditory attention, while the corpus of predictability ratings offers a resource for future benchmarking. All code and data are freely available from the R package *soundgen* and supplementary materials at https://osf.io/bgzvc.

**Supplementary Information:**

The online version contains supplementary material available at https://doi.org/10.3758/s13428-026-03153-3.

## Introduction

An important dimension along which sensory input varies is the degree to which it is predictable. This principle is particularly central to the paradigm of predictive coding, according to which top-down projections in the brain continuously attempt to match the expected input and minimize prediction errors (Friston et al., 2006; Hohwy, 2013), but predictability is relevant to many other theories of perception such as Gestalt psychology with its principle of good continuation (Kandel et al., 2021, Chapter 21). There is extensive empirical evidence that sensory stimuli involuntarily attract attention -- become more salient -- if they violate our expectations (Marsh et al., 2014; Röer et al., 2014; Sussman et al., 2003). For instance, a sound that deviates from the preceding pattern tends to “pop out” and capture our attention (Körner et al., 2019; Parmentier et al., 2022; Vachon et al., 2017; Vasilev et al., 2019). Likewise, a contextually unexpected, surprising word *banana* in the phrase “*Time flies like an arrow, fruit flies like a banana”* causes enhanced neural processing because it forces the listener to update their model of what the sentence means (Fiebach et al., 2004). Fundamentally, unexpected sensory input may be so salient precisely because it calls for re-evaluating the internal representation of the situation: where we are, who else is present, what is going on, what dangers and opportunities are present, etc. In turn, any action requires a predictive model of the environment because even simple movements like saccades have to be prepared in accordance with how the world is expected to be by the time the effectors have executed the motor command, which may be quite different from what the senses are currently reporting (Kandel et al., 2021, Chapter 30). In other words, because of unavoidable sensory and motor delays, we always have to think ahead when acting in the world, which means that anticipating sensory input and projecting to expected future states is the brain’s default mode of operation.

Given the central role of predictability in sensory processing and action planning, there has been a lot of interest in developing robust computational approaches to measuring it. Within the auditory modality, the relevant psychoacoustic research has largely been performed on sequences of simple artificial sounds, although more recent research has increasingly used more complex and natural soundscapes (Huang & Elhilali, 2017; Kothinti et al., 2021). Another major research domain is musicology, where unexpected transitions or surprising events are useful for segmenting musical pieces and understanding their affective impact on listeners (Foote, 1999; Peeters, 2023). Looking at speech processing, typical phonetic patterns are learned statistically, and surprising combinations draw attention, so surprisal and entropy play an important role in associating phonological meaning with specific phonetic details (Hume & Mailhot, 2013). Predictability is also an important aspect of conversation analysis (Angus, 2019), although speech demands a distinct analytical approach because it consists of semantically meaningful units.

In this paper, I focus on measuring the predictability of a specific type of auditory input, namely sequences of relatively stereotypical environmental sounds and vocalizations (rather than, for example, speech or music). These stimuli are simple enough to control precisely, yet biologically important and very common in everyday life, making them a good case study for the considered algorithms. From cricket chirps to human laughs, all vocal signals have to be attended to if they are to perform their communicative function, so the ability to attract attention -- salience -- is a key functional signal property in bioacoustics (Fitch et al., 2002; Ryan & Cummings, 2013). Even though adult humans mostly communicate with speech, nonverbal vocalizations like laughs and screams are also very common in everyday life (Anikin et al., 2023; Kamiloğlu & Sauter, 2024). For preverbal infants, in particular, effective vocal communication with the caretaker is absolutely critical for survival (Furlow, 1997; Zeifman, 2001). On the flip side, vocalization sequences, such as baby cries or dog barks, are often described as being very stressful and difficult to shut out even when they are irrelevant (Jégh-Czinege et al., 2020; Richey et al., 2020). Likewise, environmental sounds often constitute unwanted distractors and sources of noise pollution -- road traffic and construction noise are common examples (Bogdanov et al., 2022) -- although some may also be specially designed to draw attention, as in the case of beepers in accessible pedestrian signals or acoustic alarms in hospitals (Foley et al., 2020). In all these cases, it is important to be able to quantify the predictability of acoustic signals in order to understand the relation of bottom-up salience to attentional capture and, by extension, to the communicative effectiveness and esthetics of both natural and human-designed sounds.

In the rest of the paper, I review and benchmark several algorithms for calculating auditory surprisal and propose a new method based on autocorrelation, using a dataset of human ratings for benchmarking the performance of each surprisal measure. The objective in this paper is not to present a single off-the-shelf solution for measuring auditory unpredictability in any context, but rather (1) to review the most common approaches, (2) to explain the mathematical principles and share open-source code for implementing them, and (3) to show how each algorithm can be fine-tuned and optimized for a particular application - in this case, for approximating human judgments of the predictability of acoustic sequences.

Regarding the potentially confusing terminology, I do not make any distinction between *surprise*, *surprisal*, and *unpredictability* in this text, which all refer to unexpected events. *Surprisal* is the more technical term, but it is conventional to refer to *surprise* in certain contexts -- for instance, the extent to which prior beliefs are updated is normally called *Bayesian surprise*, not *surprisal*.

### Dataset for benchmarking

To validate and optimize the algorithms for measuring surprisal discussed below, I obtained explicit ratings of the predictability of 300 synthetic sequences of noises and animal vocalizations, each about 7 s in duration, by 195 human listeners. Synthetic sequences were used because the amount of both temporal and spectral variation could be precisely controlled. Fitted values of unpredictability per sequence from a multilevel regression model, as well as the parameters used to create the sequences, were then used for the validation and optimization of the considered algorithms.

### Stimuli

**Fig. 1:**
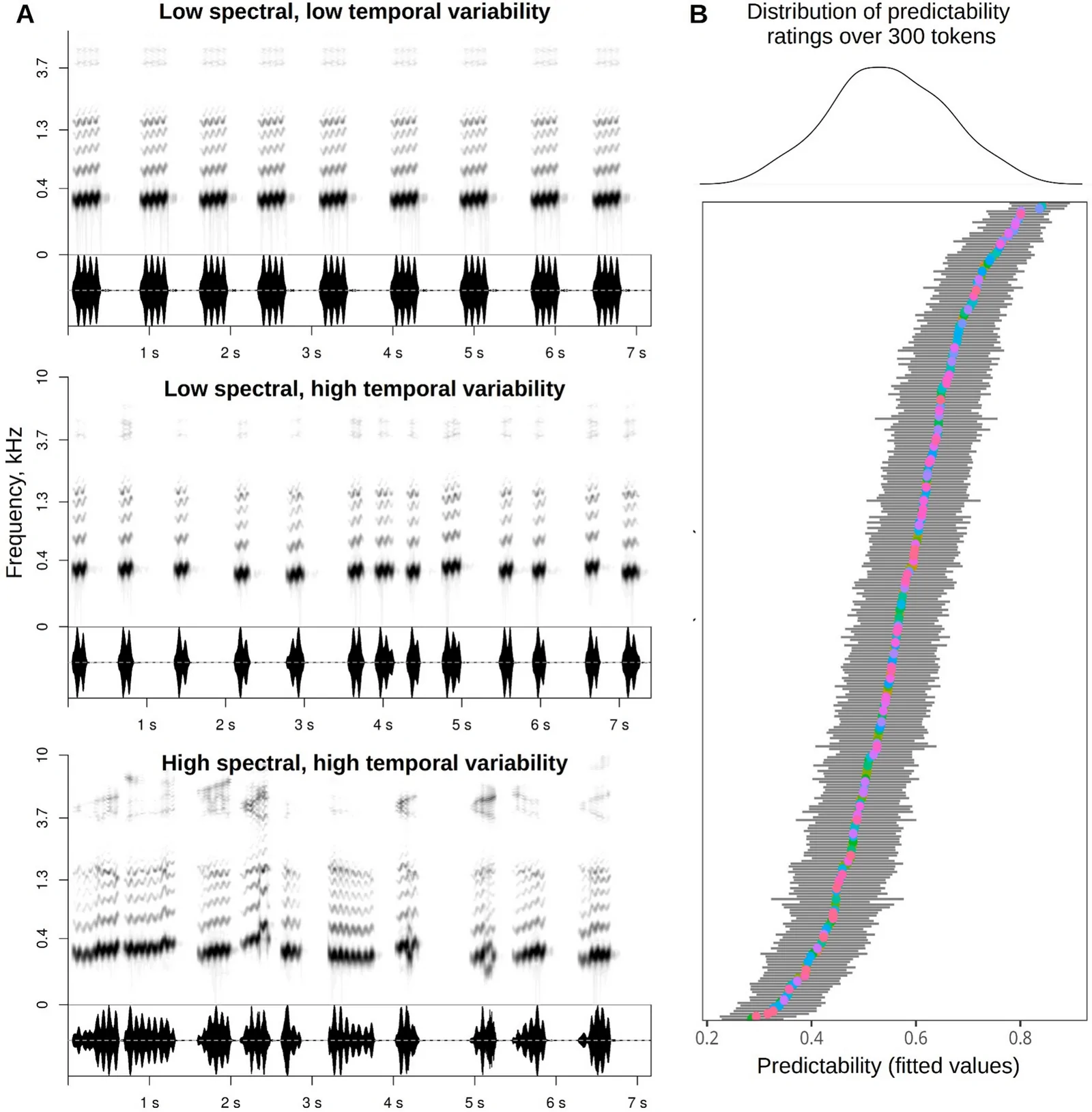
Dataset of human ratings of predictability used for benchmarking the algorithms. **A** Spectrograms showing three synthetic sequences of snipe calls with varying degrees of spectral and temporal variability. **B** The distribution of fitted values of predictability for each acoustic sequence (*N* = 300): medians of posterior distributions with 95% CIs. The *color* shows the template (*N* = 25): whistle, bark, etc

A total of 300 sequences of realistic animal calls and various noises, each ~7 s long, were synthesized with the R package *soundgen* (Anikin, 2019). They were based on 25 templates or prototypes adapted from (Anikin, 2025). Twelve tokens were created for each prototype with variable levels of stochastic variation in the irregularity of pauses between individual syllables (the *randomness* parameter) and in spectro-temporal characteristics of individual sounds or “syllables” (the *temperature* parameter; see Fig. 1A). The sequences were all normalized to have the same peak amplitude to ensure that they would be presented to listeners at comparable sound pressure levels (the subjectively experienced loudness also depends on the spectrum, individual auditory sensitivity, and playback equipment, so it would be difficult to standardize). The key acoustic variables considered in the following sections are listed in Table 1.

**Table 1:** Key acoustic variables

Variable name	Explanation
DL_audioic	Mean surprisal per sequence extracted with neural network PAAudioIC
entropy_cv	Variability of Wiener entropy of the spectrum
entropySh_cv	Variability of Shannon entropy of the spectrum
flux_mean	Mean flux of normalized acoustic features (see soundgen::analyze function)
interburst_cv	Variability of intervals between envelope peaks (irregularity of beat-like rhythmic features)
loudness_cv	Variability of subjective loudness estimated in sone units
novelty_mean	Mean SSM novelty
pauseLen_cv	Variability of pauses between individual sounds (“syllables”)
peakFreq_cv	Variability of peak frequency (the highest peak in the spectrum)
randomness	Randomness hyper-parameter used to synthesize the sequences
specCentroid_cv	Variability of spectral centroid (spectral center of gravity)
surpACF_env4	Mean ACF surprisal calculated from the envelope with a window of 4 s
surpACF_env4_onlyNeg	Same as surpACF_env4, but ignoring non-negative values
surpACF_spec1	Mean ACF surprisal calculated from a mel-spectrogram with a window of 1 s
surpACF_spec1_onlyNeg	Same as surpACF_spec1, but ignoring non-negative values
surpKL_spec1	KL divergence (Bayesian surprise) calculated from a mel-spectrogram with an analysis window of 1 s
surpSh_spec1	Shannon surprisal calculated from a mel-spectrogram with an analysis window of 1 s
sylLen_cv	Variability of the duration of individual sounds (“syllables”)
temperature	Temperature hyper-parameter used to synthesize the sequence
unpred_rat	Rating of unpredictability in the quality sample of 195 human listeners (fitted value per sound from a multilevel model)
unpred_rat_full	Same as *unpred_rat*, but using the full sample of 221 listeners

### Procedure

The perceptual experiment was written in JavaScript and performed online. Listeners rated the unpredictability of 25 sequences, including one randomly selected token of each prototype. The order of prototypes was also randomized for each participant. The question was: *How (un)predictable do you find this sequence? Are the sounds predictable (regular), in the sense that you know exactly what’s coming next?* Responses were given on a horizontal visual analog scale ranging from 1 to 100 and labeled *Not at all predictable* to *Completely predictable*.

### Participants

A total of 195 participants (115 F + 78 M + 2 unknown, age M ± SD = 43 ± 12, range [21, 84]) residing in the UK were recruited on https://www.prolific.com/. No demographic selection criteria were used because the goal was to enroll an approximately representative sample of the adult population. Age-related hearing loss can make high-frequency stimuli difficult to hear, but all manipulations were performed within the prototype, only a few of which were sufficiently high in frequency for this to be a possible issue; 92% of listeners were under the age of 60, and all self-reported having no hearing difficulties. Each of the 300 stimuli was rated an average of 16.4 ± 3.8 times with this sample size, ensuring sufficient precision of estimates. Another 26 participants were excluded because they provided incomplete submissions, reported not paying attention in the debriefing questionnaire, or provided ratings that very poorly correlated with the average ratings per token by all participants (Pearson’s *r* <.2), which is a simple heuristic for detecting low-effort responses. A sensitivity analysis confirmed that the fitted values of predictability per sound were practically identical (Pearson’s correlation *r* =.99) in the full sample (*N* = 221 listeners) and in the quality sample (*N* = 195), so excluding these 26 participants had essentially no effect on the results.

### Data analysis

The objective was to extract the population-average predictability ratings per sound. Accordingly, the following multilevel model was fit with the R package *brms* (Bürkner, 2017):

1 $$\mathrm{r}\mathrm{e}\mathrm{s}\mathrm{p}\mathrm{o}\mathrm{n}\mathrm{s}\mathrm{e} \sim 1+1\left(1\left|\mathrm{s}\mathrm{o}\mathrm{u}\mathrm{n}\mathrm{d}\right.\right)+\left(1\left|\mathrm{s}\mathrm{u}\mathrm{b}\mathrm{j}\mathrm{e}\mathrm{c}\mathrm{t}\right.\right)+\left(1\left|\mathrm{p}\mathrm{r}\mathrm{o}\mathrm{t}\mathrm{o}\mathrm{t}\mathrm{y}\mathrm{p}\mathrm{e}\right.\right), \mathrm{p}\mathrm{h}\mathrm{i}\sim \left(1\left|\mathrm{s}\mathrm{u}\mathrm{b}\mathrm{j}\mathrm{e}\mathrm{c}\mathrm{t}\right.\right)$$

The distraction ratings *R* in individual trials, which originally ranged [0, 100], were normalized to (0, 1) by taking *R*/102 + 0.01 and modeled as beta-distributed. A group-level intercept was estimated for each sound (*N* = 300), prototype (*N* = 25), and subject (*N* = 195). The dispersion parameter *phi* was also modeled for each subject to account for differences in using the response scale. Fitted values of predictability were extracted as the median values of the posterior distribution for each sound. To understand what aspects of these vocalization sequences determined listeners’ ratings, the extracted fitted values were correlated with spectro-temporal acoustic descriptives and hyper-parameters used to synthesize the sequences. Formal inferential modeling was not performed: almost any association is statistically significant with a sample size of 300 stimuli, but the precision of this analysis is greatly exaggerated because the fitted values of predictability per sound are themselves estimated with considerable uncertainty. More detailed modeling of the acoustic predictors of unpredictability at the level of individual responses is presented in a separate publication (Anikin, 2025) that used a comparable, but much larger dataset designed to test a different question, namely the effect of predictability on annoyance. Here, the focus is on the algorithms themselves, and acoustic predictors of ratings are shown merely to help with interpreting their performance. R code for replicating all analyses is available in online supplements (https://osf.io/bgzvc) as Rmd and HTML markdown documents.

### Results of the rating study

The synthesis algorithm used two hyperparameters to control sequence variability: randomness only affected rhythm, whereas temperature made individual sounds in a sequence more variable. The effect of *temperature* on unpredictability ratings (obtained from predictability by inverting the scale) was relatively consistent (*r* =.57), but *randomness* had only a moderate effect (*r* =.19). Looking at simple spectro-temporal descriptives (see Table 1 for definitions), the variability in syllable length (*sylLen_cv*) and interburst intervals (*interburst_cv*) were the best predictors of perceived unpredictability (*r* =.59 and.45, respectively), followed by the variability of purely spectral measures such as peak frequency (*peakFreq_cv*; *r* =.22) and spectral centroid (*specCentroid_cv*; *r* =.25). Thus, listeners’ ratings reflected both spectral and temporal variability in the synthesized sequences, but short-term spectral changes appeared to affect the ratings to a greater extent than irregular rhythm. In fact, some comments made by listeners at the debriefing stage suggested that they were aware of two kinds of unpredictability -- rhythmic and spectral -- and were sometimes unsure as to which one they were supposed to rate. While this distinction could be made more explicit and studied separately in follow-up research, there is value in keeping the task open-ended as it aligns more closely with ecologically relevant effects of acoustic surprisal: what kind of unpredictability the listeners choose to prioritize is informative in itself.

To ensure that the ratings would reflect gradual judgments of predictability, rather than a binary distinction between completely regular and completely irregular sequences, none of the stimuli were completely regular -- the rhythm was never perfectly isochronous, and identical sounds were never repeated, unlike in (Anikin, 2025). The perceived predictability per sound ranged from 0.31 to 0.84 on a scale of 0 to 1, estimated with an average precision of ± 0.10 -- that is, the median width of the 95% credible interval around each estimate was 0.20 (Fig. 1B). Thus, as intended, the perceived predictability of the tested acoustic stimuli ranged in a continuous manner, from very predictable to largely random.

### Algorithms

A surprising sound is difficult to predict based on the preceding input over some biologically plausible attentional window. If the objective is to model actual biological perception, the length of this analysis window is a crucial consideration, regardless of the chosen method. The immediate subjective experience of incoming sounds presumably reflects the contents of a sensory buffer also known as echoic memory, which is capable of storing, by different estimates, between a few hundred milliseconds and 1−2 s of audio in humans and other primates (Cowan, 1984; Inui et al., 2010). Of course, much longer storage is possible with attentional engagement and active re-encoding and rehearsal of processed acoustic information in the working memory (Winkler & Cowan, 2005). Nevertheless, the time scale of a few seconds could be particularly important for spontaneous detection of acoustic variation based on what we know about auditory processing in the brain.

Besides the biologically relevant time scale for predicting auditory input, the second key aspect to consider is what patterns are learned. Predictable input may come in many guises: continuous silence is perfectly predictable, but so is a steadily rising tone, noise with stochastically stable spectro-temporal characteristics, or a rhythmically pulsating beat. Even the absence of a sound can be surprising, although an unexpected deviant is generally more surprising than an unexpected omission (Baragona et al., 2025). Different approaches to quantifying the acoustic (un)predictability differ in what acoustic properties they model. I begin with a broad family of algorithms that essentially look at the distributional properties of acoustic input over time.

### Information-theoretical surprisal and Bayesian surprise

The oldest, and probably most mathematically justified, approach to quantifying the predictability of an event follows information theory, according to which events are surprising to the extent that they are improbable given some model that assigns different probabilities to different outcomes. Information-theoretical surprisal, or *Shannon surprisal*, can be defined as “a function of the input’s conditional probability given preceding context” (Zarcone et al., 2016). Specifically, surprisal *S* of an event whose predicted probability is *p* equals its negative log-probability:

2 $$\mathrm{S}=-{\mathrm{l}\mathrm{o}\mathrm{g}}_{2}\left(\mathrm{p}\right)$$

Thus defined, surprisal is synonymous with information content and closely related to information entropy, which is the expected value of surprisal -- namely, the average surprisal of all outcomes weighted by their probabilities (Huffaker et al., 2017). Surprisal as negative log-probability or its proxy is a very common measure of unpredictability of acoustic (Skerritt-Davis & Elhilali, 2019, 2021; Zarcone et al., 2016), musical (Bjare et al., 2025; Keitel et al., 2025), phonetic (Pimentel et al., 2021), and verbal (Slaats & Martin, 2025) sequences. Somewhat loosely, the term “surprisal” is also used to refer to many other conceptually related measures such as violation of model expectations in general (Skerritt-Davis & Elhilali, 2018), activation in neural networks that reflects changes in acoustic input (Bjare et al., 2025; Huang et al., 2018), etc.

A popular alternative to Shannon surprisal is Bayesian surprise, which measures the difference between prior and posterior probability distributions -- that is, between the distribution of event probabilities before and after observing the new event (Baldi & Itti, 2010; Bayram et al., 2025; Itti & Baldi, 2009; McGarry & Nelson, 2025; Schauerte & Stiefelhagen, 2013). Essentially, Bayesian surprise shows how much we update our beliefs in light of a new observation -- for instance, after observing the next value in a particular time-frequency bin in the analyzed spectrogram. Mathematically, a common way to calculate Bayesian surprise is to take the Kullback--Leibler (KL) divergence between prior and posterior probability distributions P and Q over all possible outcomes *x*, which is:

3 $${\mathrm{D}}_{\mathrm{K}\mathrm{L}} \left(\mathrm{P} \Vert \mathrm{Q}\right)=\sum\nolimits_{\mathrm{x}}\mathrm{P}\left(\mathrm{x}\right) \mathrm{l}\mathrm{o}\mathrm{g} \left(\mathrm{P}\left(\mathrm{x}\right)/\mathrm{Q}\left(\mathrm{x}\right)\right)$$

Whereas events of equal probability receive equal Shannon surprisal regardless of content, Bayesian surprise is more flexible in the sense that the information content of a rare event depends on how much it changes our prior beliefs, which is, arguably, closer to actual human reasoning (Itti & Baldi, 2009; McGarry & Nelson, 2025). The main difficulty with this approach is that we need to know not only the probability of one event, but the entire prior and posterior distributions over all outcomes, which is often computationally intractable. Fortunately, there are closed-form solutions for specific simple situations (Baldi & Itti, 2010), as well as proposed modifications for non-Gaussian distributions or even a smooth forgetting mechanism, whereby the weight of each distribution slowly decreases over time (Schauerte & Stiefelhagen, 2013). Apart from KL divergence, other proposed ways to compare prior and posterior distributions include Jensen--Shannon divergence, which is symmetric and always finite (Rodríguez-Hidalgo et al., 2018; Skerritt-Davis & Elhilali, 2021), but the general principle remains the same.

Both Shannon surprisal and Bayesian surprise are natural, theoretically justified measures, but they require assigning a probability to the observed event (e.g., a new note, word, or tone in a sequence). This probability can come from a variety of sources. For instance, a large language model like ChatGPT can return the probability that the next word in a sentence will be X, replacing traditional measures such as word co-occurrence rates. For purely acoustic input without musical or linguistic structure, the simplest and most general modeling approach demonstrated here is to compare the new observation with some distributional property of the preceding *N* observations. For instance, we can track the mean and variance of energy in a particular frequency channel over a sliding analysis window, treating normalized density of the corresponding Gaussian distribution at the next observation as a proxy for its probability and calculating measures analogous to both Shannon surprisal and KL divergence. Thus defined, Shannon surprisal *S* of observation X_t_ is calculated based on the relative probability density *ϼ*, which is a measure of the probability that X_t_ came from the same distribution as the preceding values:

4 $$\begin{array}{l}\uprho \left({\mathrm{X}}_{\mathrm{t}}\right)=\mathrm{D}\left({\mathrm{X}}_{\mathrm{t}} \left| {\mu}_{\mathrm{t}-1 },{\sigma}_{\mathrm{t}-1}\right.\right)/\mathrm{D}\left({\mu}_{\mathrm{t}-1 }\left|{\mu}_{\mathrm{t}-1}\right.,{\sigma}_{\mathrm{t}-1}\right)\\ \mathrm{S}\left({\mathrm{X}}_{\mathrm{t}}\right)=-\mathrm{l}\mathrm{o}\mathrm{g}\left(\uprho \left({\mathrm{X}}_{\mathrm{t}}\right)\right)\end{array}$$

where

D: is the probability density of a Gaussian distribution with mean = μ_t-1_ (the mean of input X over the analysis window up to time *t-1*) and standard deviation = σ_t-1_
D(μ_t-1_ | μ_t-1_, σ_t-1_): is a normalization constant equal to the maximum probability density, which ensures that *ϼ* never exceeds 1.

For Bayesian surprise, I adopted the closed-form solution proposed by (Rodríguez-Hidalgo et al. 2018) for comparing two Gaussian distributions based on their means and variances. Assuming that some acoustic feature X we are tracking is distributed as X_t-1_ ~ N(μ_t-1_, σ_t-1_^2^) at time *t-1*, and then as X_t_ ~ N(μ_t_, σ_t_^2^) at time *t*, then KL divergence between these two distributions is:

5 $${\mathrm{D}}_{\mathrm{K}\mathrm{L}}=\left({\mathrm{X}}_{\mathrm{t}} \Vert {\mathrm{X}}_{\mathrm{t}-1}\right)={\left({\mu}_{\mathrm{t} },{\mu}_{\mathrm{t}-1}\right)}^{2}/2/{{\sigma}_{\mathrm{t}-1}}^{2}+\left({{\sigma}_{\mathrm{t}}}^{2}\left|{{\sigma}_{\mathrm{t}-1}}^{2}-1-\mathrm{l}\mathrm{o}\mathrm{g}\right.\left({{\sigma}_{\mathrm{t}}}^{2}/{{\sigma}_{\mathrm{t}-1}}^{2}\right)\right)/2$$

It may be preferable to take the logarithm of KL divergence (Rodríguez-Hidalgo et al., 2018) so as to reduce its spikiness. As long as the data-generating process remains constant, Shannon surprisal does not depend on the analysis window length, but KL divergence drops as the overlap between two compared distributions increases, and this effect can be approximately canceled by adding twice the logarithm of the analysis window length to log-KL. This approximation works best if the analysis windows are sufficiently long (in practice, about 100 points; Fig. S6). It has no effect on the KL contour over time, but helps to standardize absolute KL values across analyses performed over different time scales. To make the model more biologically plausible, a “window of forgetfulness” can also be added to both Shannon surprisal and Bayesian surprise by calculating weighted means and variances, where the weights are taken from a half-Gaussian taper normalized to sum to 1. With this modification, observations more than half an analysis window in the past make a rapidly diminishing contribution to the calculation of surprisal.

#### Optimization of information-theoretical surprisal

The extraction of information-theoretical surprisal can be optimized based on Pearson’s correlation of mean Shannon surprisal per sound (*N* = 300) with human ratings of unpredictability. Except when using amplitude envelope instead of a spectrogram, performance is somewhat better without log-transforming the input, regardless of the number of frequency channels (Fig. S1). Mel-warped short-time Fourier transform (STFT) spectrograms perform best with long windows (100 ms and above, with a step of 10–20 ms; Fig. S2), and they offer both faster execution and better performance than auditory spectrograms with the same number of frequency channels and temporal resolution obtained by bandpass-filtering the signal (Fig. S3). Increasing the number of frequency channels beyond ~20 does not noticeably change the results, though, of course, higher temporal resolution is always preferable given enough computational resources.

**Fig. 2:**
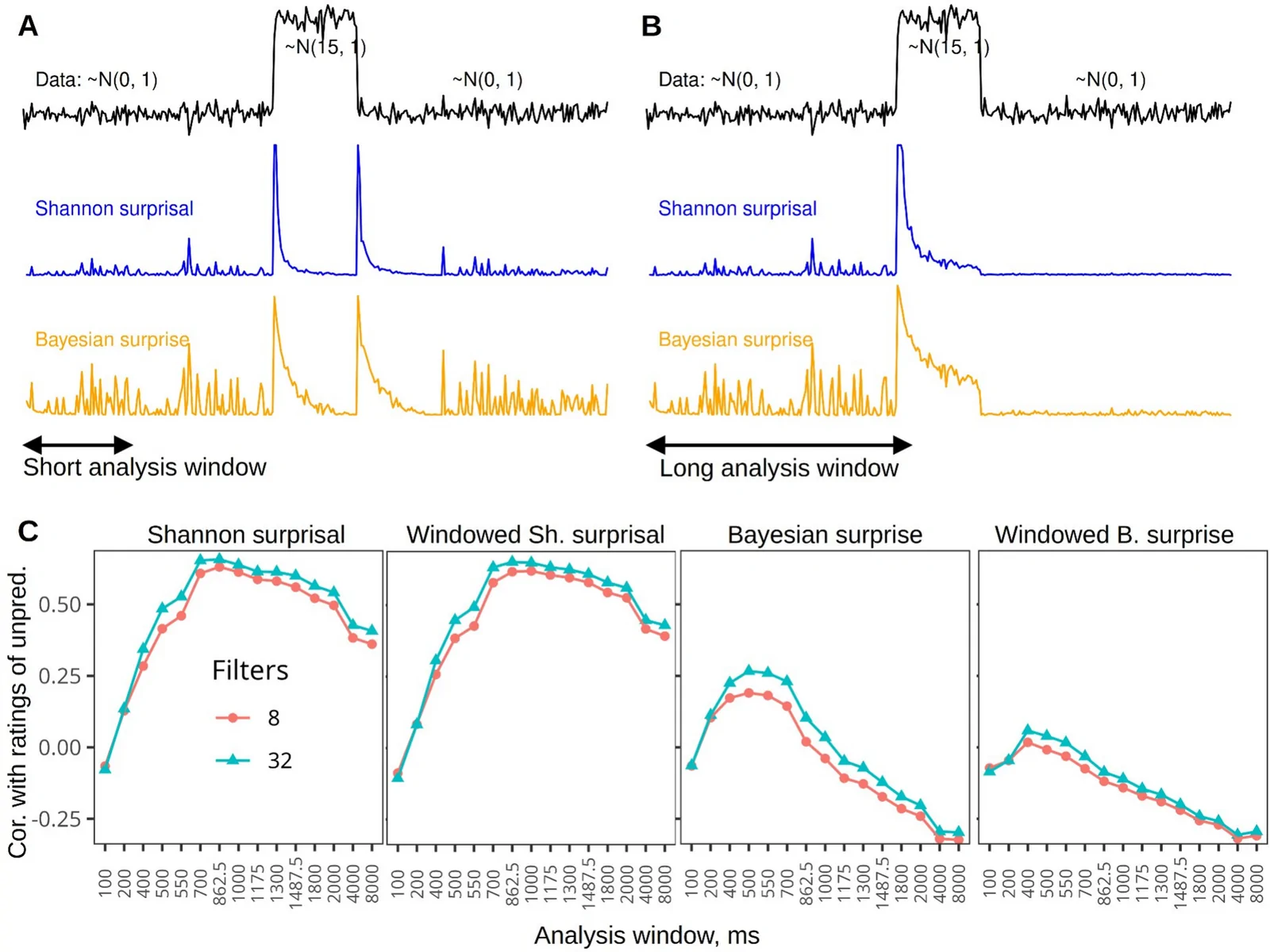
Information-theoretical surprisal. **A, B** Surprisal calculated from a univariate time series with values taken from two different normal distributions. Surprisal peaks at each transition between episodes when the analysis window is short relative to the length of an episode. With a longer window, the transition back to the original distribution is not surprising. **C** An analysis window of ~1 s is optimal for reproducing human ratings. Optimization with mel-spectrograms using a 100-ms STFT window and a step of 20 ms using either 8 or 32 mel-frequency bands (filters)

The main parameter of interest is the duration of the analysis window over which surprisal is calculated (not to be confused with the STFT window length). This key parameter determines the time scale of the analysis (Fig. 2A, B). Its optimal duration, which gives a correlation of *r* >.6 with human ratings, lies between ~700 and 1000 ms for the unwindowed version of Shannon surprisal (Fig. 2C) and is a bit longer for the windowed version, which is natural given that windowing emphasizes the contribution of more recent values. Bayesian log-surprise peaks at slightly shorter analysis windows (~500–700 ms), but it is less closely associated with human ratings (*r* =.25 with optimal settings; Fig. 2C).

#### Discussion of information-theoretical surprisal

The proxy measure for Shannon surprisal, as calculated here, reflects the probability that the current amplitude in a frequency band belongs to the same Gaussian distribution as the preceding amplitudes over some time period (e.g., over the preceding 1 s). This is a proxy rather than true Shannon surprisal because it is calculated from normalized Gaussian probability densities rather than actual probabilities of acoustic events, but with this assumption, surprisal is straightforward to compute in closed form for any arbitrary input. Despite its simplicity, mean Shannon surprisal per sequence proved to be a good predictor of the human ratings of its overall unpredictability. Surprisingly, the closely related Bayesian surprise (KL divergence) in this case was a much poorer predictor of perceived unpredictability. This is curious because the contours of Shannon surprisal and Bayesian log-surprise within a sequence were usually very similar (*r* >.9 on a frame-by-frame basis), indicating that both measures picked up on the same acoustic events as surprising. A different implementation of Bayesian surprise or a different measure of divergence than the log-KL divergence used here might perform better, which can be investigated in future studies. There is also the separate question of which measure is better at detecting brief unexpected events capable of triggering attentional capture, which is different from measuring the overall perceived predictability of entire acoustic sequences.

### Autocorrelation: ACF surprisal

Distributional approaches reviewed above will flag a sudden change in sound quality, but they do not model rhythm-like (ir)regularity at all and remain blind to recurrence patterns. Time dependencies can be taken into account by modeling covariances between successive observations with D-variate Gaussian distributions (Skerritt-Davis & Elhilali, 2019), but here I focus on two simpler alternatives. The first is to check the autocorrelation function (ACF), and the second is to compute a self-similarity matrix.

A signal is said to be autocorrelated if it repeats itself to some degree. The best-known acoustic example is the fundamental frequency, which in voice research corresponds to the rate at which the vocal folds oscillate, and more generally to the lowest frequency component of a harmonic tone, which is usually perceived as its pitch. The same concept can be applied at much longer time scales: for instance, imagine that a dog is barking at relatively regular intervals. Provided that we consider a bout long enough to include several barks, the peak of its autocorrelation function will correspond to the typical call rate -- say, 3 Hz, or three barks per second. If the interval before the next sound deviates from this typical rate -- say, if the dog barks too late or too early -- or if the call comes at the expected time but is spectrally different from the preceding ones, the bout’s autocorrelation function (ACF) will change; specifically, the peak at the identified fundamental period of 1/3 s will become lower. This constitutes a surprising event. On the contrary, if the signal is repeated perfectly -- if the dog barks at exactly the expected time and the new bark is acoustically similar to the preceding ones -- the peak autocorrelation at the fundamental period of 1/3 s will be reinforced, which can be thought of as “negative surprisal”.

This simple mathematical intuition is formalized in the function *getSurprisal* from the *soundgen* package. It transforms audio input into a spectrogram-like representation -- for instance, an auditory spectrogram with several bandpass filters representing hair cells in the cochlea, with biologically plausible central frequencies (e.g., on an ERB or bark scale) and bandwidths, although a mel-warped STFT spectrogram leads to similar results. The envelope of each filter (frequency band) is discretized at a particular sampling rate, and then a sliding analysis window is applied to the resulting spectrogram. For each time *t*, the ACF of the envelope of a frequency channel is calculated, and the lag at its highest peak is considered to be the fundamental period *T*. The value of ACF at time *t-1* and lag *T* (AC_t-1_) is treated as a measure of periodicity over the analysis window. *T* can be calculated separately for each frequency channel or forced to be the same for all channels within the analysis window to focus on the overall rhythm across all frequencies (boolean argument *sameLagAllFreqs*). Optionally, the ACF and surprisal values can also be weighted by the maximum amplitude per channel -- not RMS amplitude or energy because we expect silences between calls -- so as to give priority to louder, and therefore more perceptually salient, frequency components (argument *weightByAmpl*). The autocorrelation is then recalculated at the same lag *T* after adding the next time-frequency value (AC_t_). ACF surprisal at time *t* and frequency *f* is calculated as the change in peak autocorrelation at the same lag *T*, weighted by the length of the analysis window *L* and, optionally, by the absolute value of AC_t-1_ (argument *weightByPrecision*). Thus, for each frequency channel *f*, we have

6 $${\mathrm{S}}_{\mathrm{t},\mathrm{f}}=\left({\mathrm{A}\mathrm{C}}_{\mathrm{t}-1,\mathrm{f}}-{\mathrm{A}\mathrm{C}}_{\mathrm{t},\mathrm{f}}\right)\times \mathrm{L} \times | {\mathrm{A}\mathrm{C}}_{\mathrm{t}-1,\mathrm{f}}|$$

Multiplying by *L* accounts for the fact that a single new observation will have a diminishing impact on the ACF as the length of the analysis window increases. As for multiplying by the modulus of AC_t_, it is essentially a prior: if no pattern is apparent (|AC_t_| ~ = 0), any new value is going to be less informative for a given change in ACF compared to a new observation that causes a change of similar magnitude in the ACF of an initially highly regular signal. In the terminology of predictive coding, this is similar to the idea that the brain’s responses to deviant stimuli reflect precision-weighted prediction errors: the higher our prior certainty in the current predictive model, the more surprising we find a new observation that is incompatible with it (Lecaignard et al., 2022; Zhao et al., 2025).

If no peak is found in the ACF, *S*_*t,f*_ can either be treated as undefined or calculated using the highest absolute value of the ACF, which usually leads to choosing a lag of 1 (zero lag is excluded because it simply corresponds to the correlation of a signal with itself, which is always 1). The same argument that controls this behavior (*onlyPeakAutocor*) also determines whether surprisal should be calculated when the signal X is static over the analysis window and the ACF is undefined, namely as:

7 $${\mathrm{S}}_{\mathrm{t},\mathrm{f}}=\left|\left({\mathrm{X}}_{1,\mathrm{f}}-{\mathrm{X}}_{\mathrm{t}+1,\mathrm{f}}\right)\right./\left({\mathrm{X}}_{1,\mathrm{f}}+{\mathrm{X}}_{\mathrm{t}-1,\mathrm{f}}\right)|$$

S_t,f_ can theoretically be any real number, although in practice values over ±1 are rare (~1% of analysis frames in the examples studied here), and a simple logistic transform (argument *rescale*) can make it stay strictly within (-−1, 1). To obtain the overall surprisal at time *t*, its values are averaged over all frequency channels -- optionally, weighting by maximum amplitude in each channel (argument *weightByAmpl*).

**Fig. 3:**
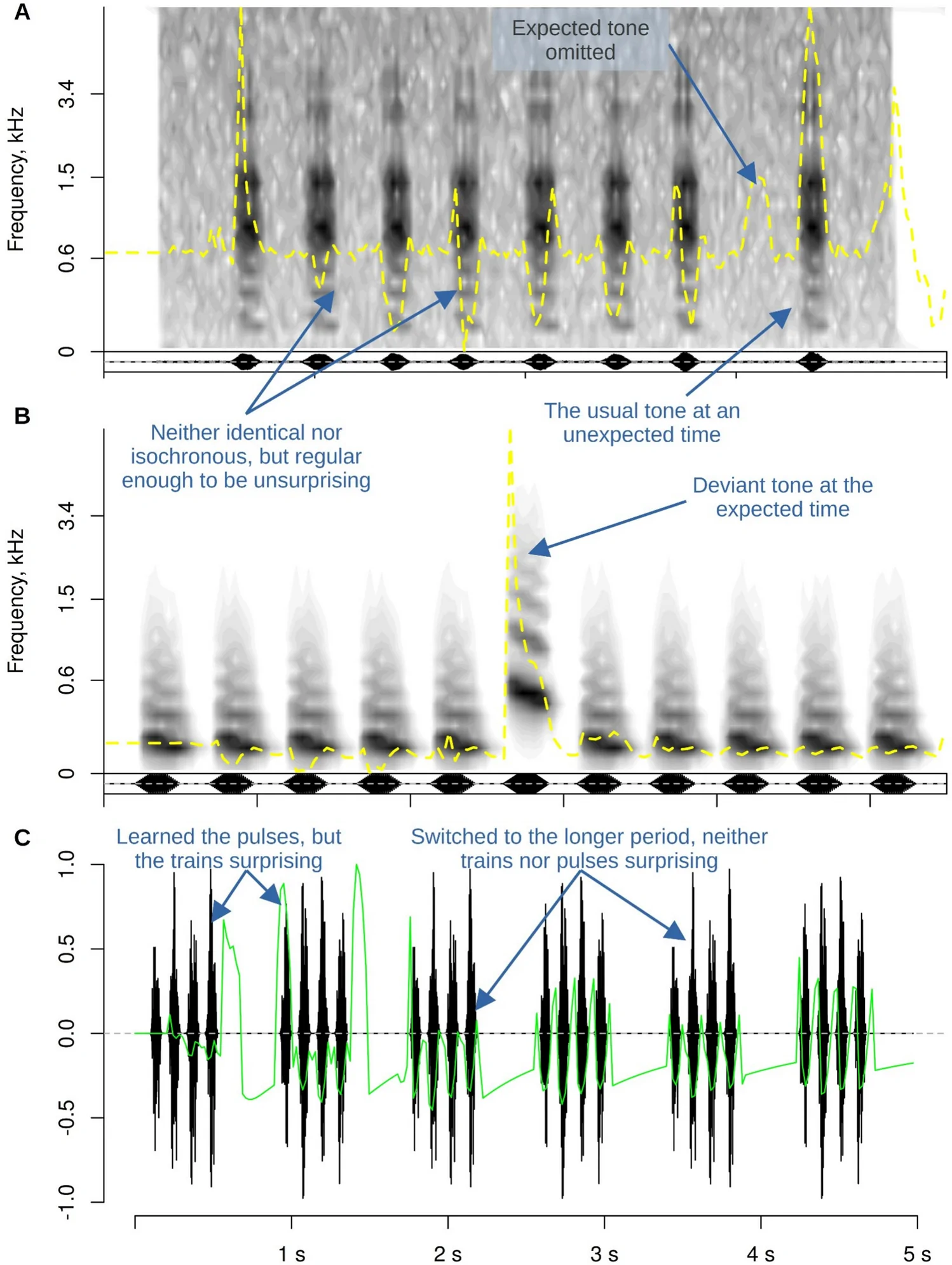
Extraction of ACF surprisal with the function *soundgen::getSurprisal()*

Just as autocorrelation-based pitch detection is robust to deviations from perfect periodicity (Boersma, 1993), ACF surprisal detects quasi-periodicity in biologically realistic, noisy sequences of sounds. For instance, the rhythm in Fig. 3A is not perfectly isochronous, and the sounds are not identical; nevertheless, after a few approximate repetitions, the pattern is learned and ceases to be surprising until a sound is omitted, whereas other tested algorithms do not find this unexpected omission surprising (Fig. S4). Although autocorrelation measures how closely a pattern is repeated, ACF surprisal is not merely a measure of rhythmic regularity: it captures both temporal and spectral (un)predictability. Thus, a deviant tone at the expected time is as surprising as a tone of the expected frequency at an unexpected time (Fig. 3B). The analysis can be performed at different time scales and thereby reveal nested regularities. In the example shown in Fig. 3C, for instance, the faster periodicity of pulses initially controls the surprisal contour (*T* = 125 ms), but the ACF switches to the slower rhythm of pulse trains (*T* = 325 ms) after observing the first two trains. Either of these two time scales can be chosen explicitly by setting the appropriate duration of the analysis window (*winSurp*).

#### Optimization of ACF surprisal

**Fig. 4:**
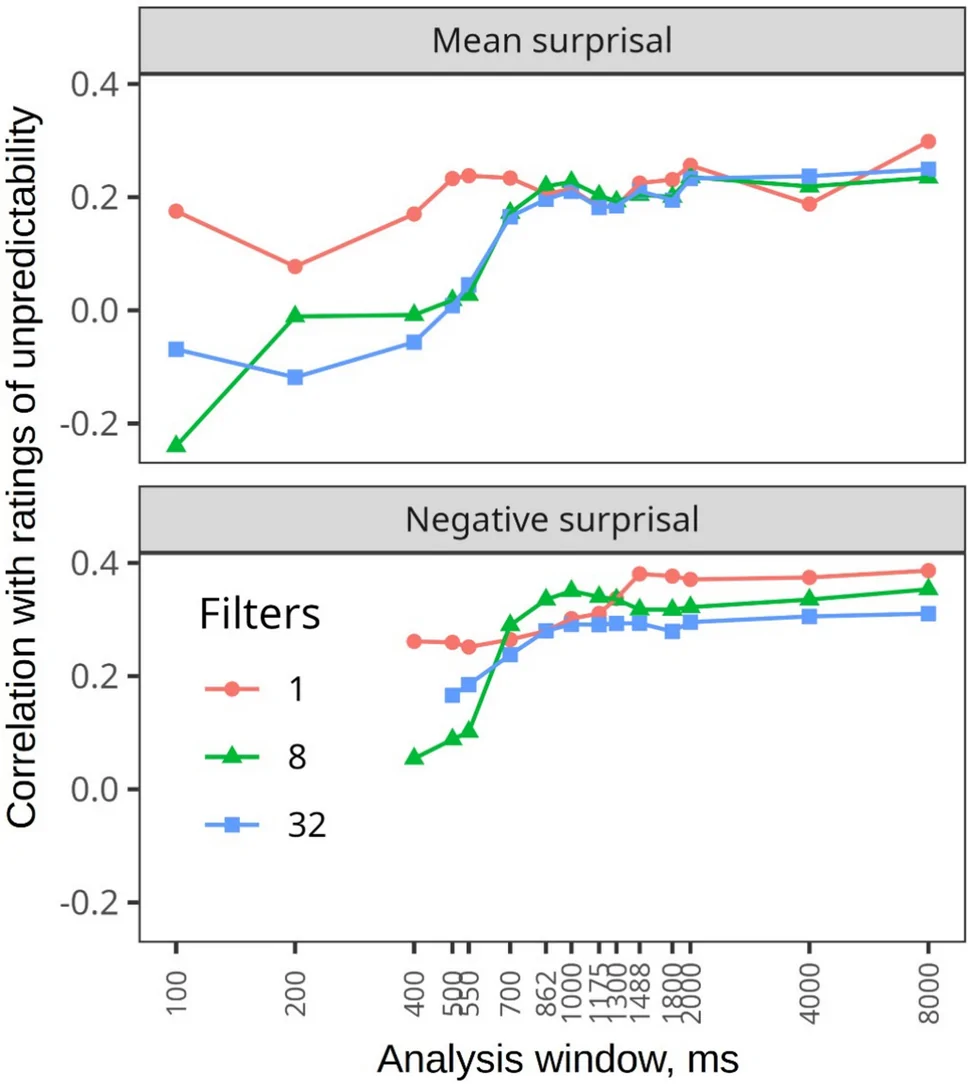
Optimization of ACF surprisal: the effect of analysis window duration and the number of frequency bands (filters). ACF surprisal has a natural zero; the *upper panel* shows correlations between human ratings and the global mean of all surprisal values per sound (positive and negative), whereas in the *lower panel* only negative values of surprisal are considered

Many of the settings in the algorithm can be set a priori, based on biological plausibility. For instance, it is natural to use log-spectrograms (or log-envelopes) because loudness perception is basically logarithmic (Fastl & Zwicker, 2006), although in practice log-transforming the input has little effect on the results. Similarly, weighting by channel amplitude does not make a dramatic difference, and the results are also robust with respect to the type of spectrogram representation (e.g., the number of filters and temporal resolution). Working with simple amplitude envelopes (*nFilters* = 1) is very fast and sufficient for analyzing purely rhythmic unpredictability. Of course, computing a full spectrogram is essential for detecting spectral deviants, but the results stabilize quickly with just 8 or 16 frequency channels, at least with the sounds considered here (Fig. 4).

The key setting is the length of the analysis window (*winSurp*). Mean ACF surprisal per sequence begins to correlate with human ratings of unpredictability as the analysis window increases to about half a second and plateaus above ~700 ms at a low level of Pearson’s *r* ~ =.2. Interestingly, this low correlation is due to opposite trends for the positive and negative parts of the surprisal contour. The negative part of surprisal shows higher correlations with unpredictability ratings, particularly when surprisal is weighted by precision (up to *r* ~ =.4; Fig. 4). Thus, sequences with low dips into negative surprisal (i.e., with repeated patterns that reinforce the ACF) are rated as more predictable. The proportion of surprisal contour that is positive is also positively correlated with unpredictability ratings, but the mean positive surprisal tends to be *higher*, not lower, in the sequences rated as more predictable (see *optim_surprisal.html* in online supplements for details).

ACF surprisal is a very flexible measure that can be fine-tuned to capture various recurrent patterns in acoustic sequences. Longer windows of about 4 s are good for capturing rhythmic (un)predictability (as evidenced by the improved correlation with the *randomness* hyper-parameter; see Fig. 7), and for this purpose it is enough -- in fact, even preferable -- to consider just the envelope instead of obtaining a spectrogram. Shorter windows of about 1 s applied to an auditory spectrogram with at least about 8 frequency bands are suitable for capturing spectral variability (as evidenced by the correlation with the *temperature* hyper-parameter and other measures of spectral variability; see Fig. 7 and Fig. S5).

#### Discussion of ACF surprisal

To understand what ACF surprisal is really measuring, it is important to distinguish between its positive and negative components. Information-theoretical surprisal is unipolar in the sense that, whatever the scale of its values, it fundamentally ranges from low to high surprisal. In contrast, ACF surprisal is bipolar with a natural zero: positive values indicate a deviation from the preceding pattern (a drop in peak autocorrelation at the lag corresponding to the fundamental period), whereas negative values indicate a reinforcement of the preceding pattern (a boost of peak autocorrelation). These two components behave differently depending on the settings, and there may be a tradeoff between measuring them optimally. With the settings chosen here (especially with regard to weighting by precision), negative surprisal is optimized for capturing spectro-temporal predictability, whereas the average value of positive surprisal is higher for the sequences that are rated more predictable, not less. This happens because relatively regular sequences build up strong expectations in the form of high autocorrelation (high precision), following which even a relatively minor deviation from the pattern registers as highly surprising, causing a brief, but pronounced spike in ACF surprisal, particularly if it is weighted by precision. A similar phenomenon is sometimes reported in neuroimaging studies: if brain responses are prediction errors, we would expect them to decrease when a surprising stimulus becomes more predictable, but sometimes the opposite is the case. This may be because even a small deviation becomes very surprising when precision is high (e.g., if we have been listening to a sequence for a long time) (Lecaignard et al., 2022; Zhao et al., 2025). Based on the present results, however, it is not entirely clear whether or not weighting by precision helps to approximate human perception: it does for recurrence (negative surprisal), but not for deviations from it (positive surprisal).

A possible objection is that there is no need to use ACF surprisal to measure what is essentially irregular rhythm because the same information is available “for free” from simple temporal descriptives such as the variability in call rate or inter-beat intervals (e.g., variable *interburst_cv*). There are two reasons why ACF surprisal is more generally applicable. First, it can capture recurrent spectral patterns that are not reflected in the temporal structure based on the signal’s envelope alone, such as a deviant tone that obeys the rhythmic expectations (Fig. 3B). Second, the stimuli analyzed here consist of individual sounds separated by silences, but in many situations it may not be possible to segment a sequence so neatly, or the beat may be buried in the background noise and only visible in certain frequency bands.

The distinction between positive and negative ACF surprisal is not merely a complication: it provides more flexible analytical options compared to unipolar surprisal scales. Specifically, average negative ACF surprisal can be seen as a measure of long-term spectrotemporal regularity, whereas peaks in positive surprisal mark locally deviant acoustic events. Importantly, benchmarking in this study was performed on the average perceived unpredictability of entire sequences, and in this task recurrence patterns (negative surprisal) mattered more than their occasional violations (positive surprisal). However, local prediction errors are known to be highly salient (Röer et al., 2014; Sussman et al., 2003), and peaks in positive ACF surprisal can be used to detect such locally deviant sounds. As with any surprisal measure, the analysis can also be performed on multiple time scales: for instance, rhythmic regularity can be assessed with an analysis window of several seconds using only amplitude envelopes, whereas local spectral deviants can be detected with a shorter analysis window of about 1 s and using spectrograms as input (see Table 2 for recommended settings).

**Table 2:** Key optimized surprisal measures and their extraction

Measure	Code (soundgen v2.9.0)	Speed*	What it detects
Shannon surprisal, Bayesian surprise	getSurprisal(path, winSurp = 1000, input = 'melspec', melfcc_pars = list(nbands = 20, windowLength = 100, step = 20), takeLog = FALSE)	x7 (x10 without ACF)	Sudden changes in the spectrum
ACF surprisal, short analysis window with spectrogram as input	mean_onlyNeg = function(x) mean(x[x < 0], na.rm = TRUE) getSurprisal(path, winSurp = 1000, input = 'audSpec', audSpec_pars = list(nFilters = 8, step = 15, minFreq = 60), takeLog = TRUE, sameLagAllFreqs = FALSE, weightByAmpl = TRUE, onlyPeakAutocor = TRUE, rescale = FALSE, summaryFun = c('mean', 'mean_onlyNeg'))	x7	Short-term spectrotemporal recurrence (negative part) and its violations (positive part)
ACF surprisal, long analysis window with envelope as input	getSurprisal(path, winSurp = 4000, input = 'env', env_pars = list(step = 15), takeLog = TRUE, sameLagAllFreqs = FALSE, weightByAmpl = TRUE, onlyPeakAutocor = TRUE, rescale = FALSE, summaryFun = c('mean', 'mean_onlyNeg'))	x35	Long-term rhythmic recurrence (negative part) and its violations (positive part)
SSM novelty	ssm(path, input = 'melspec', takeLog = FALSE, melfcc_pars = list(windowLength = 125, step = 25, nbands = 50), kernelLen = 1000, sparse = TRUE)	x15	Transitions between spectrally homogeneous fragments

*Execution speed on 4 cores of i7-8550U, seconds of audio/second of machine time

### Recurrence: SSM novelty

**Fig. 5:**
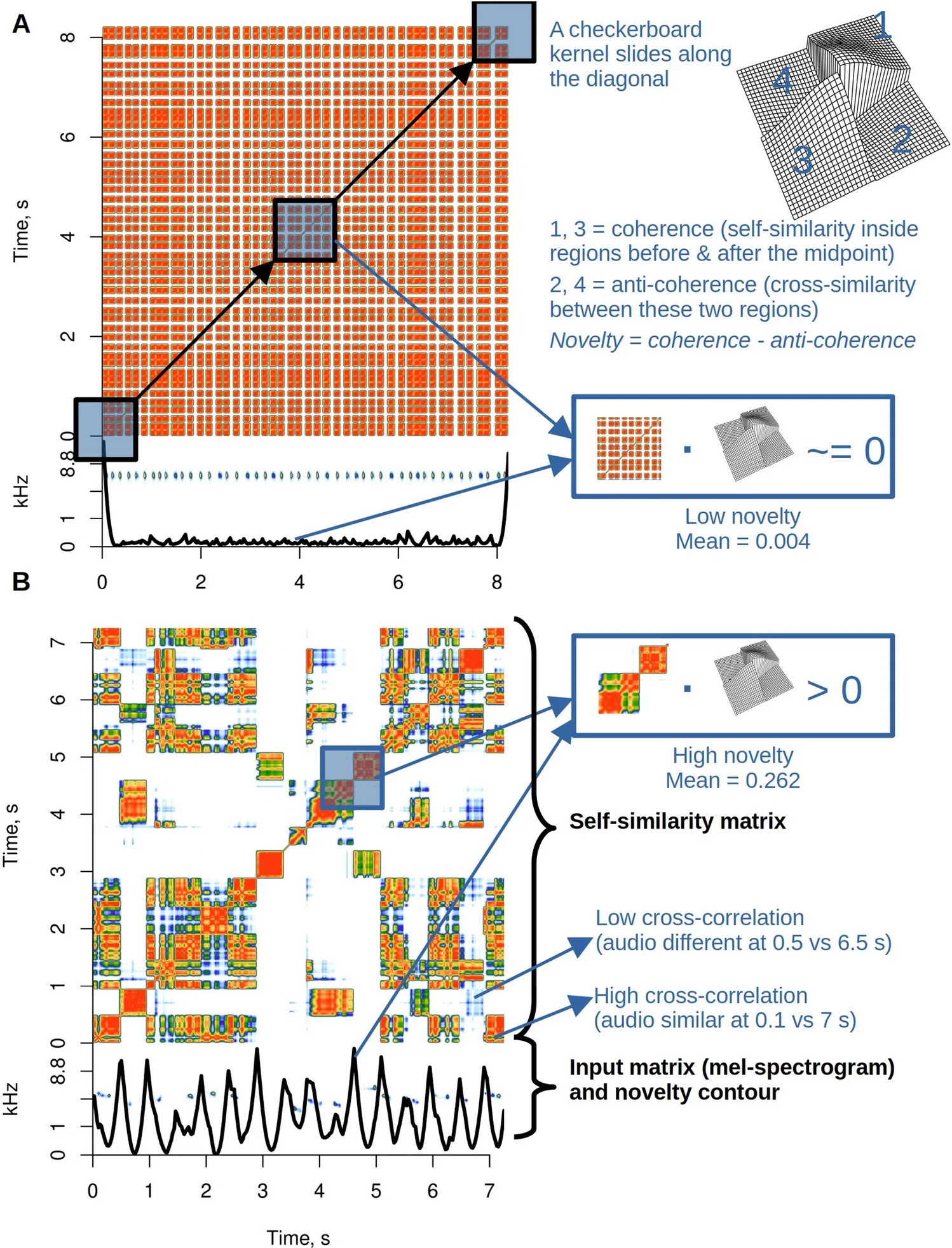
Novelty calculation from self-similarity matrices. **A** This low-novelty example consists of a single short, high-frequency chirp repeated at approximately equal intervals. Each chirp is visible in the SSM as a small red square, and the mean novelty of this sound is 0.004. **B** This high-novelty example consists of longer chirps that are very variable in frequency and rhythm. The checkerboard kernel is 1 s long (40 STFT frames), so the cross-correlated regions reach up to ±500 ms from each analyzed point. The mean novelty of this sound is 0.262

Besides autocorrelation, another way to analyze recurrence patterns is to look at the self-similarity matrix (SSM) obtained by cross-correlating segments of a recording. SSMs were first proposed to visualize musical structure (Foote, 1999), but the general idea is much older: recurrence plots are a classic technique in nonlinear time series analysis (Huffaker et al., 2017). To obtain an SSM, each short segment of audio is converted to a vector of features (e.g., mel-frequency cepstral coefficients or a column of a spectrogram) and cross-correlated with all preceding and following frames, resulting in a symmetric square matrix with time along both dimensions, where the main diagonal corresponds to a lag of zero (Fig. 5). This representation is not merely a visualization tool: it can be used to segment a recording into relatively homogeneous fragments by convolving the SSM with a checkerboard kernel sliding along the main diagonal; a Gaussian taper is added to the kernel to avoid edge effects. The positive lobes of the kernel represent coherence (self-similarity within the regions on either side of the center point) and the negative lobes anti-coherence (cross-similarity between these two regions). Since novelty is the dot product of the checkerboard kernel with the SSM, it is high when the two regions are self-similar (internally consistent) but different from each other (Foote, 2000). Like ACF surprisal, this method is fundamentally based on correlation, but here we compare two adjacent segments of a recording instead of checking how well a single new value fits into a previously observed pattern.

Apart from its original application to music structure, various types of recurrence plots are also used to analyze the rhythm and hierarchical structure of animal vocalizations (Burchardt & Knörnschild, 2020; Malige et al., 2021) and the recurrence of topics in conversation analysis (Angus, 2019). SSM novelty has also been successfully applied to processing a variety of data, including non-acoustic time series such as measurements from biosensors (Rodrigues et al., 2022). The method is very general because any time series data can be used as input: not just spectrogram-like representations, but any auditory features, inter-onset intervals in rhythmic sequences, semantic vectors, etc.

#### Optimization of SSM novelty

**Fig. 6:**
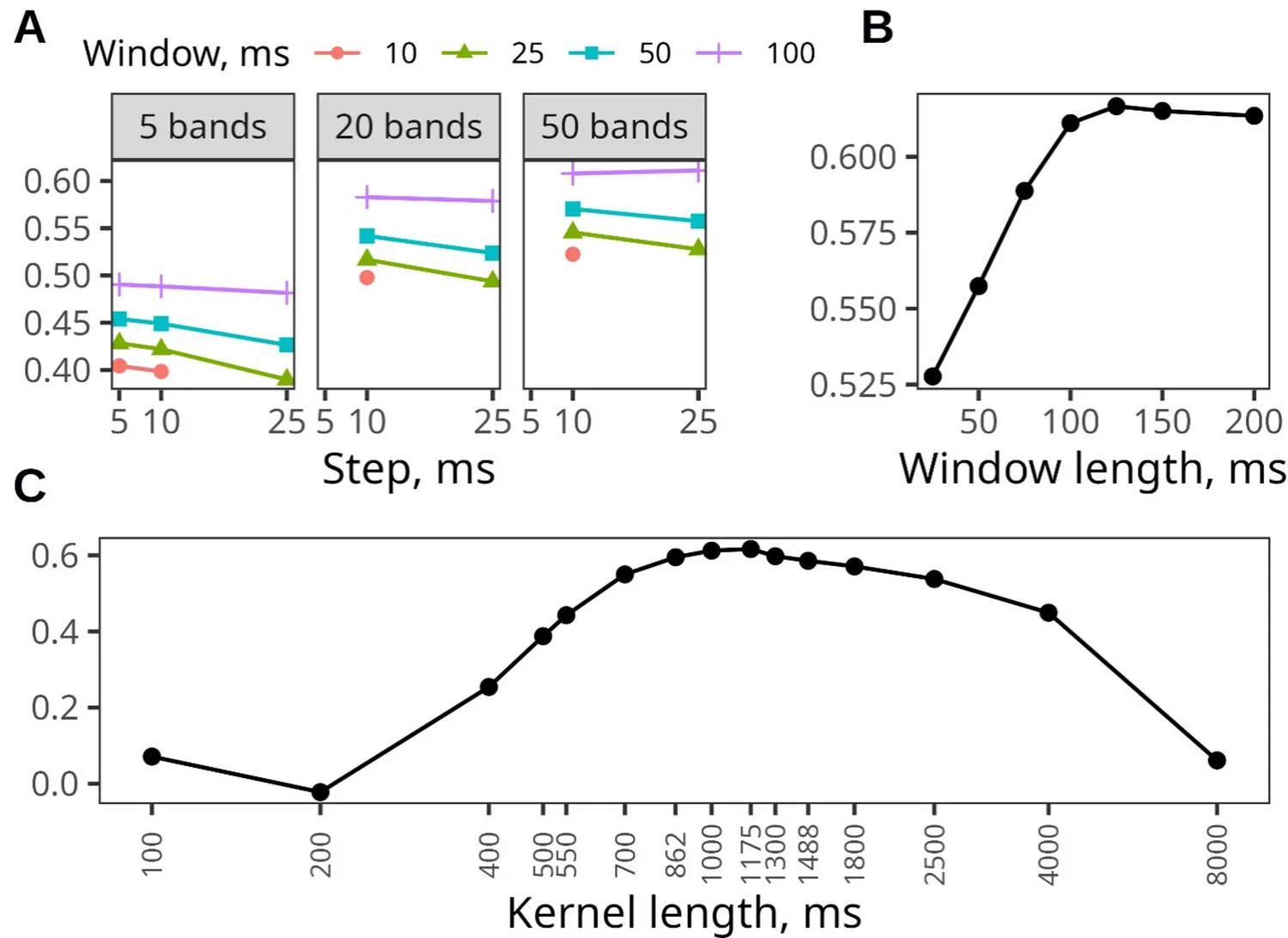
Optimization of SSM novelty. **A** Optimizing STFT window length and step as well as the number of frequency bands (filters) in mel-spectrograms using a kernel length of 1175 ms. **B** A more detailed look at the effect of STFT window length with a kernel of 1175 ms, a step of 25 ms, and 50 mel-frequency bands. **C** The effect of kernel length with an STFT window of 125 ms, a step of 25 ms, and 50 mel-frequency bands. All *Y*-axes show Pearson’s correlation with human ratings per sound

The choice of spectrogram type has little effect on the results (mel-frequency cepstral coefficients, mel-warped STFT spectrogram, or auditory spectrogram), but mel-warped spectrograms are attractive because of their transparency and computational efficiency. Rather long STFT windows of 100 ms or even more and with several dozen mel-frequency bands offer high frequency resolution and produce results that are most similar to human ratings of predictability (Fig. 6A, B). The most relevant parameter by far is kernel length, with optimal performance achieved in the range of about 900 to 1800 ms (Fig. 6C). Thus, novelty is best calculated over a window of ±450–900 ms around the center point.

#### Discussion of SSM novelty

While recurrence plots are reasonably well known, SSM novelty has remained rather obscure outside engineering work on music segmentation. Yet, it may have a large untapped potential as an efficient way to measure the unpredictability of biological signal sequences on a particular time scale -- for instance, in order to estimate their bottom-up salience. In an earlier publication, I showed that SSM novelty predicted the ratings of distraction caused by human nonverbal vocalizations (Anikin, 2020), and the present results confirm its suitability for measuring auditory surprisal.

Curiously, SSM novelty was never intended to model actual attention: it was developed as a purely engineering solution for segmenting audio recordings. In fact, the algorithm may appear to be biologically implausible because it needs to peek into the future to work. Information-theoretical surprisal and ACF surprisal are based on estimating the probability that a new observation deviates from the pattern observed over some preceding window. Observations beyond the time window of “forgetfulness” do not affect the result, nor do the algorithms consider future values, so they can be implemented to work in real time. In contrast, SSM novelty is calculated from the recurrence patterns over an entire recording, and novelty at time *t* depends on acoustic characteristics over time *t ± w/2*, where *w* is the duration of the checkerboard kernel. However, as long as *w* does not exceed some plausible limits of short-term memory, there is no reason why two audio fragments could not be mentally compared, yielding a retrospective novelty-like surprisal estimate with a delay of *w*/2. There is no evidence that the brain actually does this, but it is not impossible.

### Neural networks

Neural networks are not necessarily an alternative to the algorithms described above, but rather a very powerful and flexible component to integrate in the analysis. The most straightforward application is to use a pretrained model to generate probabilities of transitions, which can then be used to calculate Shannon or Bayesian surprise. For instance, this approach was utilized to show that surprising phones (Pimentel et al., 2021) tend to be longer in duration. Surprisal was defined as log-probability of finding a phone in a particular context, where the probability was returned by a network trained to predict the next sound from preceding ones. Similarly, Bjare et al. (2025) used autoregressive transformer networks trained on 64-dimensional latent representations (Music2Latent) instead of raw audio, and log-probabilities of one-step predictions served as a proxy for surprisal. Even if surprisal per se is not calculated, prediction errors from neural networks constitute a measure of unpredictability. For instance, this method was used to show that intonation patterns were less predictable in infant-directed speech than in adult-directed speech (MacDonald et al., 2020).

In addition to generating probabilities to plug into information-theoretical measures of surprisal, neural networks can be used to transform raw audio into feature vectors (e.g., embedding in semantic space for linguistic analyses) or to optimize particular processing stages. Thus, deep learning can help to optimize both the calculation of self-similarity and the shape of checkerboard kernels for extracting SSM novelty (Peeters, 2023). Even if the network is not trained to represent anything related to predictability or surprisal, the amount of fluctuation in the activity of high-level layers tracks changes in the acoustic scene over time and is sometimes used as a proxy for surprisal (Huang et al., 2018). Finally, neural networks can be trained to predict human perception of surprising acoustic events such as unexpected transitions between musical pieces. Apart from explicit judgments or labeling of surprising acoustic events, training data can include physiological measurements such as EEG (Brodbeck et al., 2018; Huang et al., 2018; Lecaignard et al., 2022; Skerritt-Davis & Elhilali, 2021; Zhao et al., 2025) or pupil dilation (Zhao et al., 2022).

**Fig. 7:**
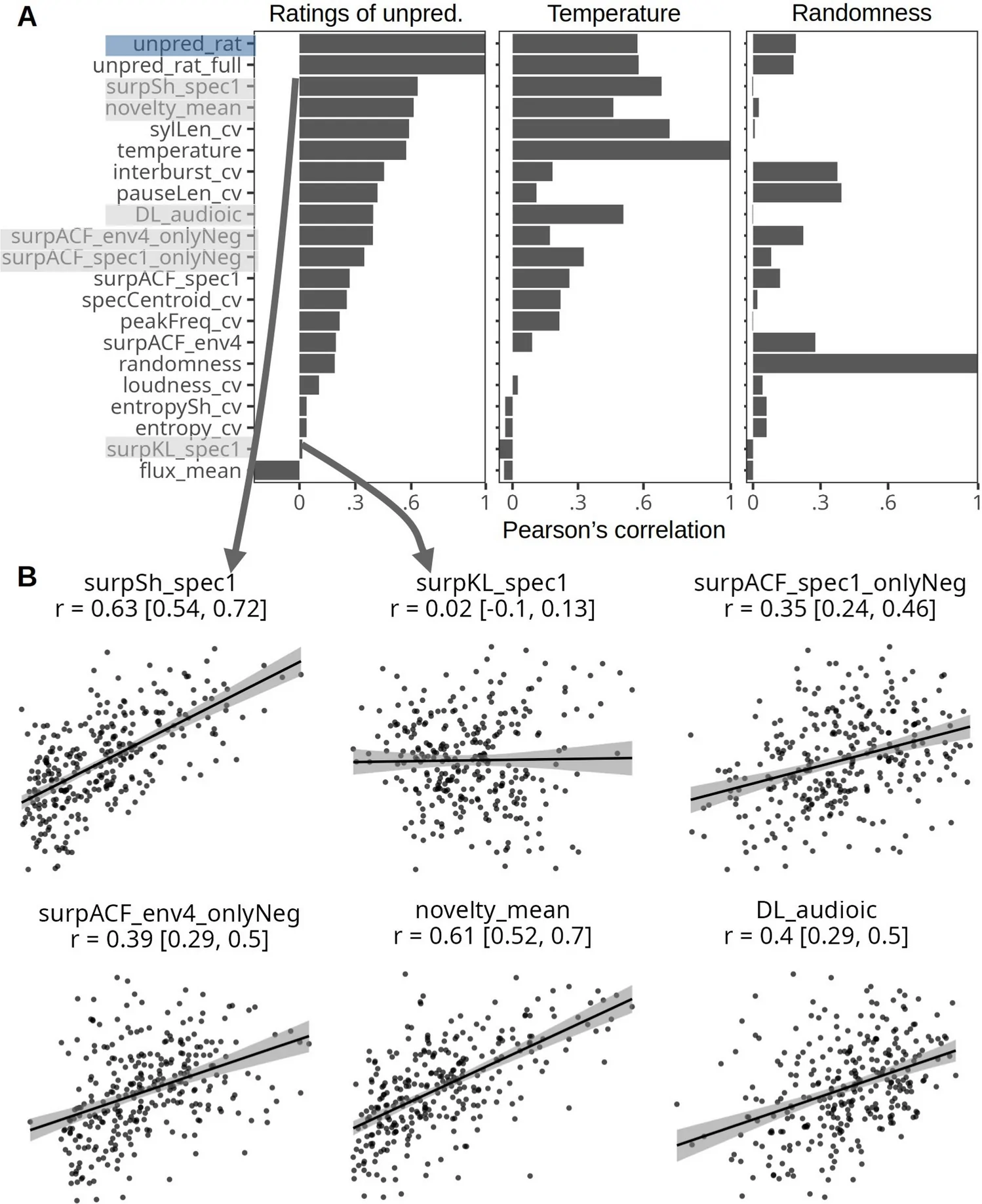
Summary of acoustic correlates of perceived and actual unpredictability. **A** Correlations with “ground truth” parameters. Note that all surprisal-related parameters were extracted with settings optimized for this specific dataset, so their correlations with human ratings are exaggerated due to overfitting. CV = coefficient of variation (SD/mean). **B** Scatterplots of six selected measures of surprisal (*highlighted with gray* in panel A) with human ratings of unpredictability per sound (*N* = 300), with correlations and 95% CIs taken from Bayesian linear regression. The recommended optimized settings and R code for extracting these surprisal measures are given in Table 2

The use of neural networks in audio segmentation and surprisal estimation is an active area of research, and its complete review goes beyond the scope of this paper. As an illustration of the sort of algorithms that are rapidly becoming available, I analyzed the same corpus of 300 acoustic sequences with PAAudioIC, an open-source Python package for extracting surprisal contours from music (Bjare et al., 2026). The mean value of surprisal obtained with default PAAudioIC settings (variable *DL_audioic* in Fig. 7) achieved a correlation of *r* =.40 with human ratings and was closely related to both Shannon surprisal (*surpSh_spec1*) and the *temperature* hyper-parameter (Fig. S5), suggesting that it mostly tapped into short-term spectral variability. Thus, even though PAAudioIC was designed for a different task -- namely, for tracing the surprise that people experience while listening to music -- it also captures some acoustic characteristics relevant to judgments of the predictability of sound sequences.

A summary of all features discussed above (Table 1) is presented in Fig. 7 in terms of their relation to three measures treated as “ground truth”: human judgments of predictability and two hyper-parameters that were used to generate the stimuli, of which *randomness* controlled rhythmic irregularity and *temperature* made individual sounds more variable. Since in this case human judgments align more closely with variation in individual sounds in a sequence than with their rhythm, good predictors of the *temperature* parameter also tend to correlate strongly with the ratings (e.g., Shannon surprisal and SSM novelty), but rhythm-related measures (e.g., variability in interburst intervals or ACF surprisal with a long analysis window) could well prove more useful with acoustic input that evokes a stronger perception of beat -- for example, music.

Last but not least, various measures of unpredictability can be combined into a single index. For instance, simply adding up three surprisal measures -- Shannon surprisal, SSM novelty, and ACF surprisal with a 4-s window, normalized to put them on the same scale -- produces a combined index that is more closely related to human ratings than either measure alone (*r* =.72). Adding two temporal measures - variability in syllable length and in interburst intervals -- improves the result further (*r* =.78). In other words, different algorithms for estimating unpredictability capture different perceptually relevant aspects of auditory surprisal, and combining them is a natural way to approximate human judgments more closely.

## General discussion

In this paper, I show how several algorithms can be adapted for estimating the overall perceived unpredictability of sound sequences by averaging over contours of locally surprising acoustic events. I conclude by discussing some implications and limitations of the present findings.

The choice of dataset for benchmarking is bound to affect the apparent performance of different algorithms. The stimuli tested here were 300 sequences of animal calls, such as dog barks or human screams, and of various environmental noises. While fairly general and ecologically relevant, this corpus only covers one specific type of auditory input -- namely, sequences of sounds that are relatively stereotypical. Music, speech, and less structured soundscapes with overlapping sound sources may well call for a different approach to quantifying acoustic surprise. For instance, just because recurrence plots are useful for analyzing the predictability of vocalization sequences does not mean that they would also be suitable for detecting salient acoustic events in continuous background noise recorded in a cafeteria. Furthermore, the settings for each algorithm were chosen based on their performance within this one sample of 300 recordings, without cross-validation, which risks overfitting. The precise settings are also likely to be sensitive to the type of input and to the task. For instance, the analyzed synthetic recordings were free from background noise and trivial to segment into separate sounds separated by silences, which is seldom the case in more naturalistic soundscapes. However, the general principles behind each algorithm are very general, and each can be adapted and fine-tuned to suit a wide variety of tasks and research applications. In particular, the described algorithms do not require any special preprocessing or segmentation of audio recordings prior to the analysis.

A second limitation pertains to the chosen “ground-truth” measure of predictability, which was derived from explicit ratings of the average perceived predictability of entire sequences by human listeners. Popular alternatives to this approach are to focus on the detection of particularly salient acoustic events or to track how surprised listeners feel over time, and explicit judgments can be supplemented with a variety of implicit indicators such as EEG responses (Bayram et al., 2025; Bjare et al., 2025) or pupil dilation (Fleischmann et al., 2025). It is important to reiterate that the reported performance benchmarks and optimized settings are specific to the chosen task. The purpose of this paper is not to find the surprisal measure that is optimal for all applications, but rather to explain how several algorithms work, share open-source code for implementing them (see Table 2 for a summary), and show how they can be adapted and fine-tuned flexibly.

Despite these limitations, there are two noteworthy findings to highlight. First, the algorithms presented here are not redundant: they tap into different aspects of spectro-temporal unpredictability and therefore produce complementary measures of surprisal. Thus, understanding the principles behind each measure of surprisal can help to select the most appropriate algorithm and fine-tune it for a specific task. Specifically, SSM novelty and spectral-distributional Shannon surprisal are more sensitive to short-term spectral changes, making them suitable for detecting local spectral deviants. In contrast, ACF surprisal and temporal measures derived from syllable segmentation or beat detection can also capture rhythmic irregularity on longer time scales. Because the resulting surprisal estimates are only weakly correlated and, presumably, reflect different perceptual processes, combining them into a single index can also improve performance in terms of approximating human perception. On a methodological note, algorithms that focus on spectral variability (SSM novelty, Shannon surprisal) benefit from improved frequency resolution that can be achieved with longer STFT windows or more filters in auditory spectrograms. In contrast, autocorrelation-based surprisal is conceptually related to beat perception and does not require high frequency resolution even if the input is a spectrogram rather than just an amplitude envelope.

The second take-home message is that most optimized measures of surprisal converge on a time scale of about 1 s (from about 700 ms to 1500 ms) as the most relevant for estimating spectral variability. This aligns closely with the estimated duration of pre-attentional echoic auditory memory (Cowan, 1984; Inui et al., 2010), which is consistent with the interpretation that the extracted measures capture some of the biological reality of processing auditory information in the brain. Of course, longer time scales may be useful for capturing slower acoustic changes such as the (ir)regularity of slow rhythms, but even rhythm perception is subject to memory constraints. Slow beats with inter-onset intervals of over 1 s are increasingly difficult to follow without explicitly paying attention, and the beat is no longer perceived as such if it is sufficiently slow (Bååth et al., 2016). Thus, temporal irregularities on time scales that exceed the capacity of echoic and working memory are probably less likely to be noticed and therefore less relevant for modeling auditory perception. As a final word of caution, none of the presented algorithms is intended to serve as a standalone model of attention, or even of bottom-up auditory salience. Neurocognitive models of attention comprise bottom-up and top-down auditory processing, separation of sound sources and scene analysis, goal setting, etc. (De Coensel et al., 2009; Huang & Elhilali, 2020; Kothinti et al., 2021; Shamma et al., 2011), and the predictability of acoustic input discussed in the present work is only one aspect of this much broader problem.

## Supplementary Information

Below is the link to the electronic supplementary material.

Supplementary file1 (PDF 2202 KB)

## Data Availability

All data and materials are available at https://osf.io/bgzvc.

## Code availability

All data and materials are available at https://osf.io/bgzvc. This includes R code for running all reported analyses. The functions for measuring surprisal are included in the open-source R package *soundgen,* freely available from https://cran.r-project.org/package=soundgen.
