# Supplementary material for: Measuring surprisal in sound sequences
Source: Behav Res Methods. 2026 Aug 24;58(10):278. doi: 10.3758/s13428-026-03153-3 (PMC13503466; doi:10.3758/s13428-026-03153-3)
Supplement: Supplementary file 1 [file 13428_2026_3153_MOESM1_ESM.pdf]

Supplementary Tables and Figures

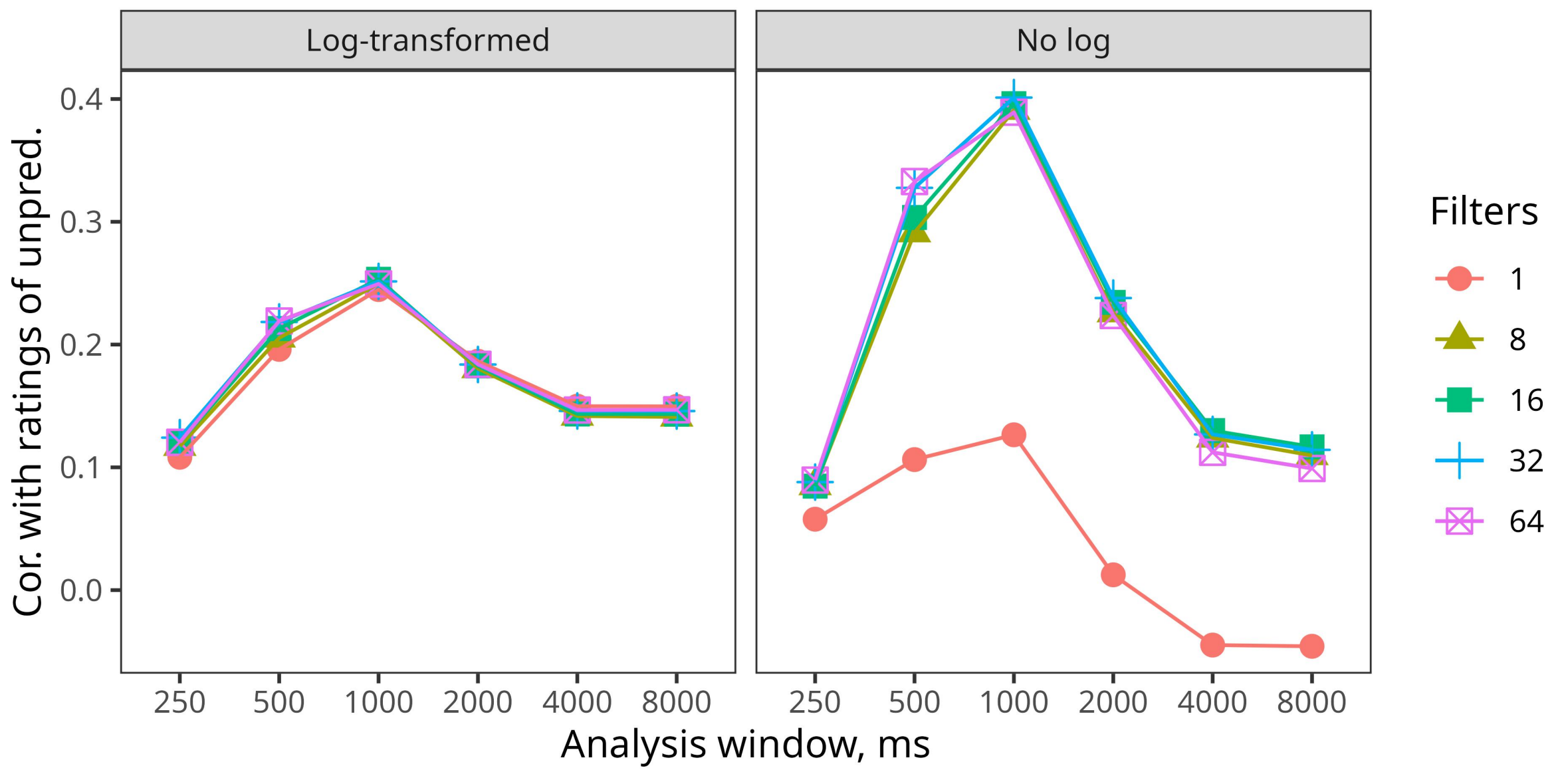

Fig. S1 Optimization of Shannon surprisal: the effect of analysis window and log-transforming the input (mel-warped STFT spectrograms with a window length of 40 ms and a step of 20 ms). Using a single filter means extracting an amplitude envelope rather than a spectrogram.

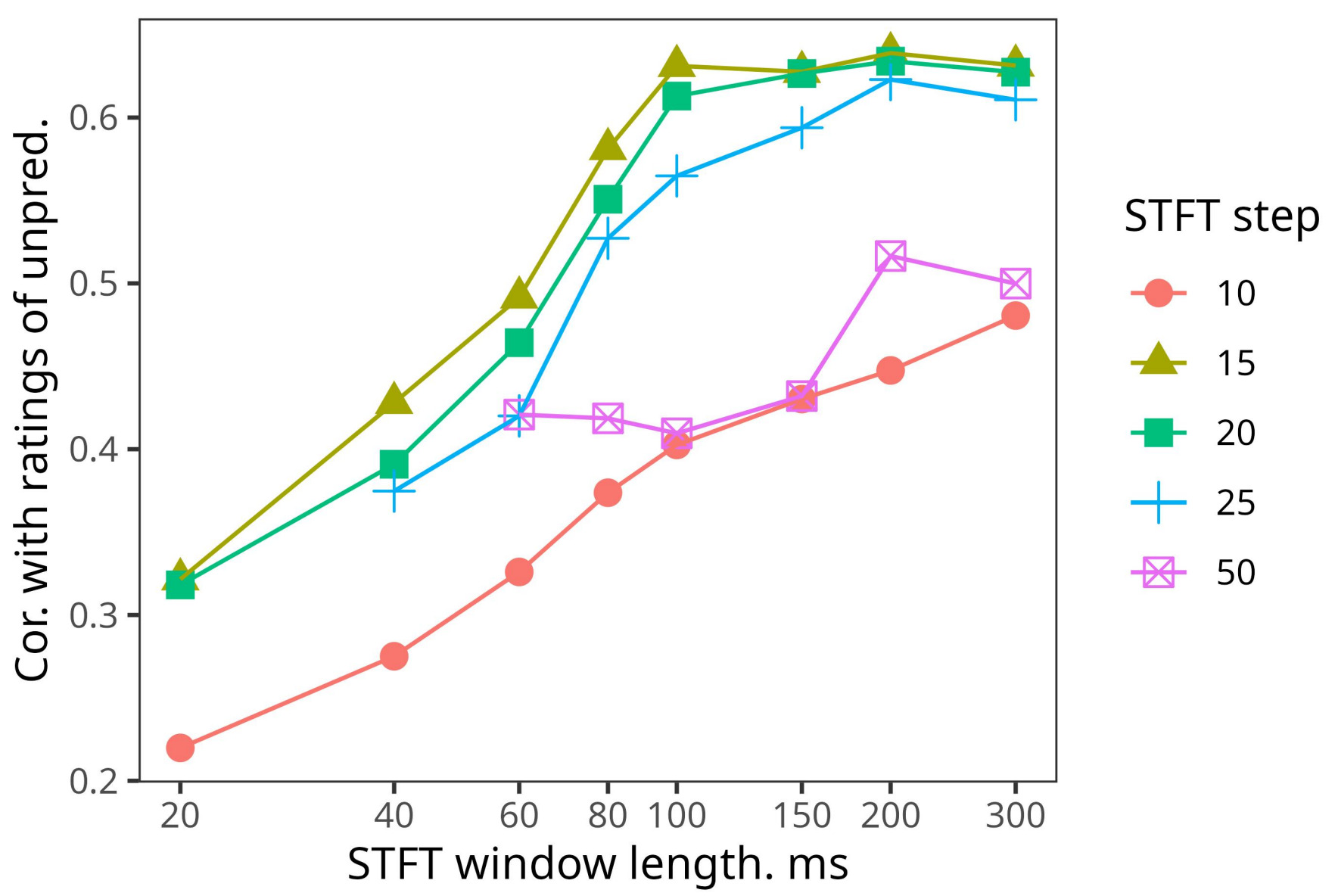

Fig. S2 Optimization of Shannon surprisal: the effect of STFT window length and step. A 1-s analysis window was used throughout; the auditory spectrograms were produced with 8 frequency channels and were not log-transformed.

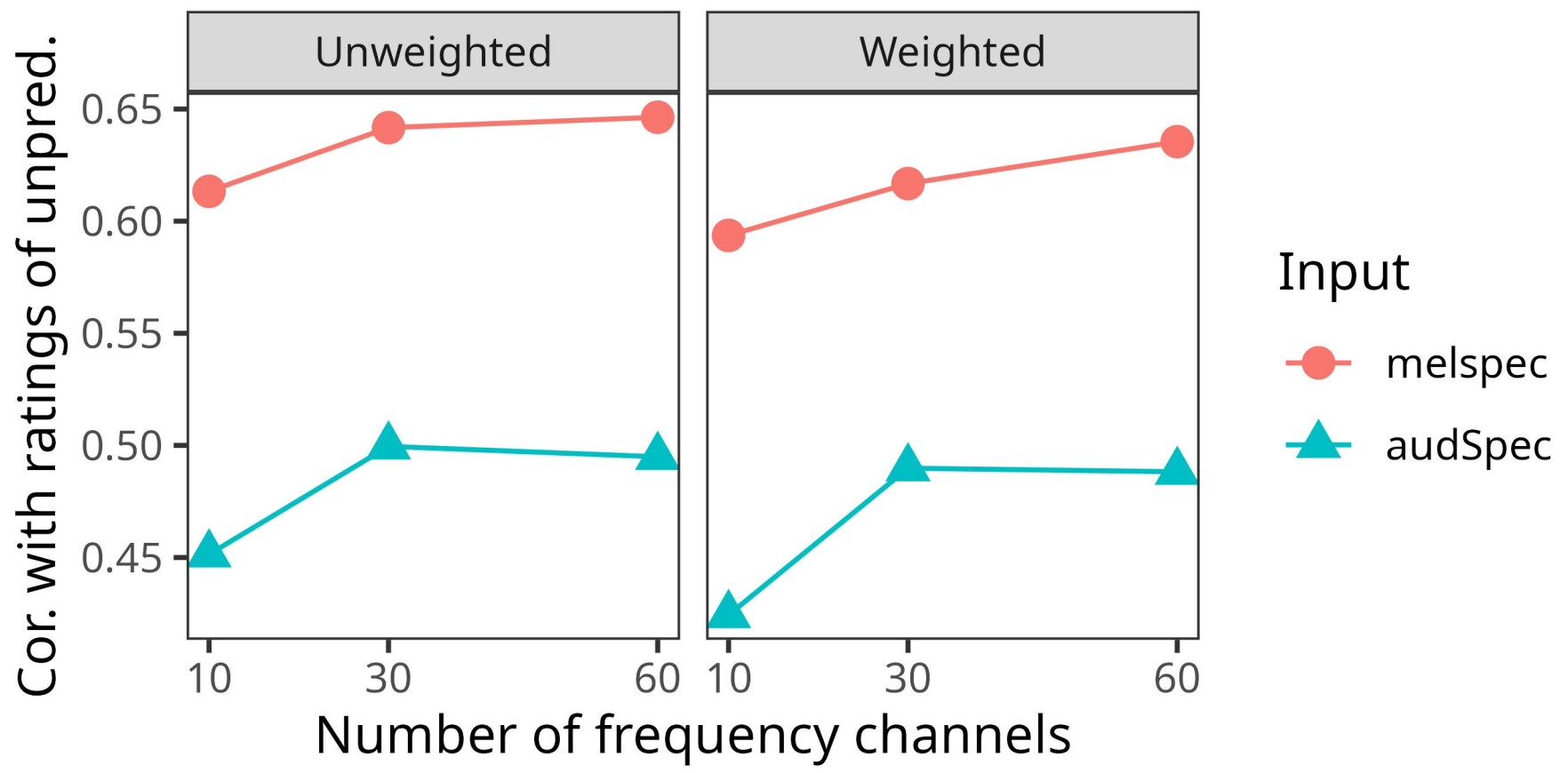

Fig. S3 Optimization of Shannon surprisal: the effect of spectrogram type and weighting by maximum amplitude per channel in each analysis window. A 1-s analysis window was used throughout; Mel-spectrograms were produced with an STFT window of 125 ms and a step of 25 ms; auditory spectrograms used a step of 25 ms.

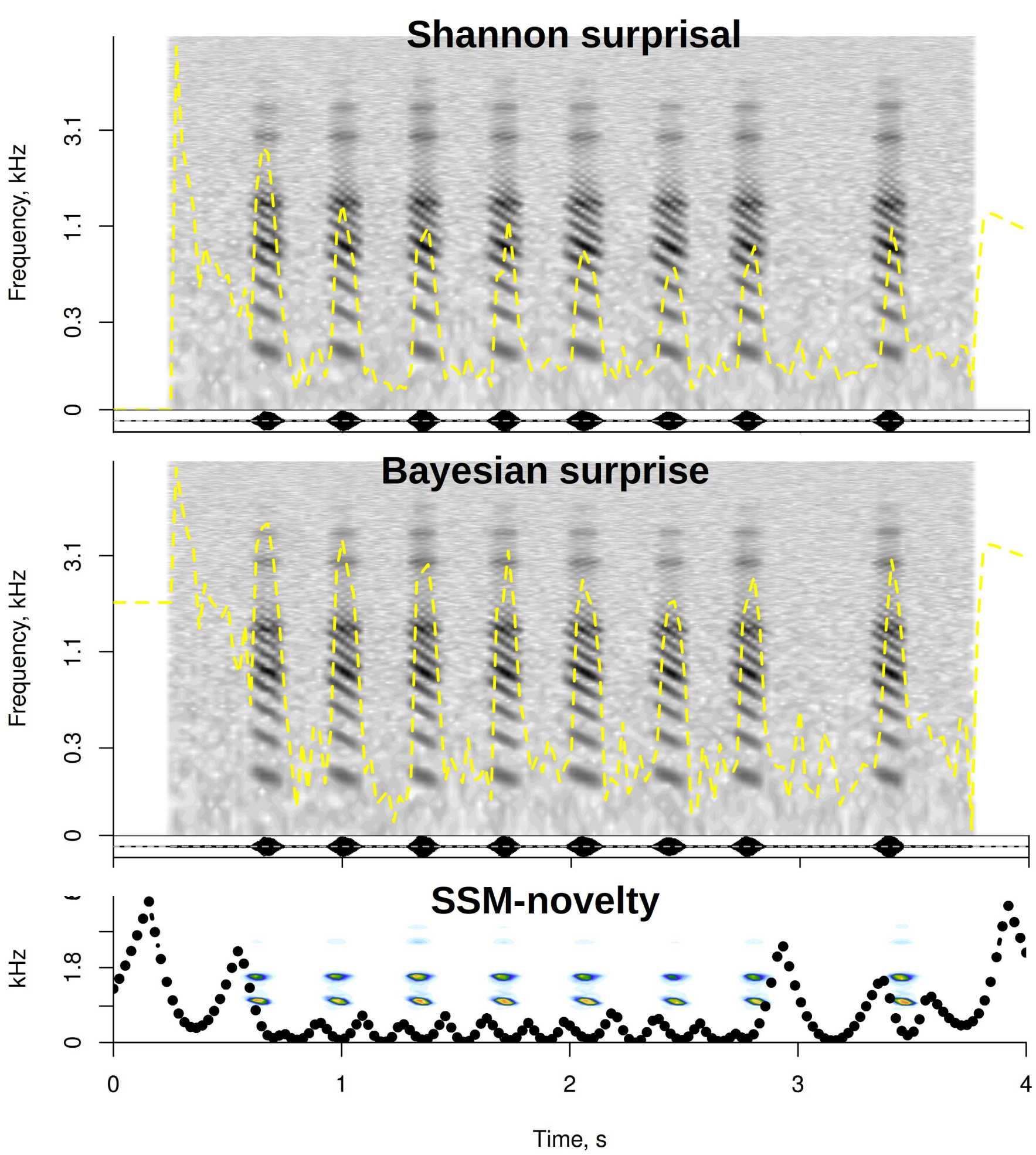

Fig. S4 Re-analysis of the example in Fig. 3A. Other measures of surprisal fail to pick up the regular patterns tracked by ACF-surprisal.

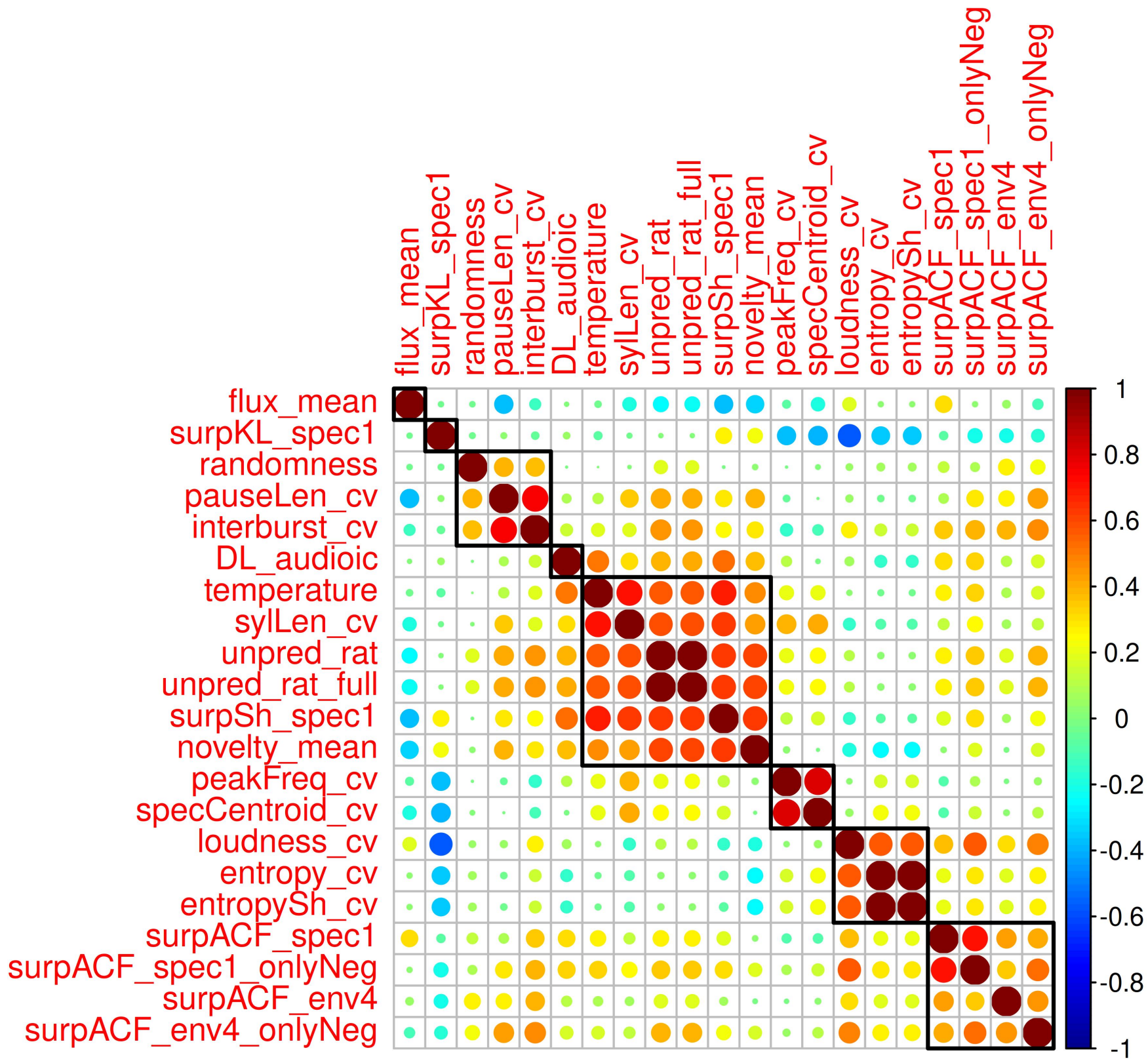

Fig. S5 Correlations between the acoustic features in Fig. 7, ordered and grouped with hierarchical clustering.

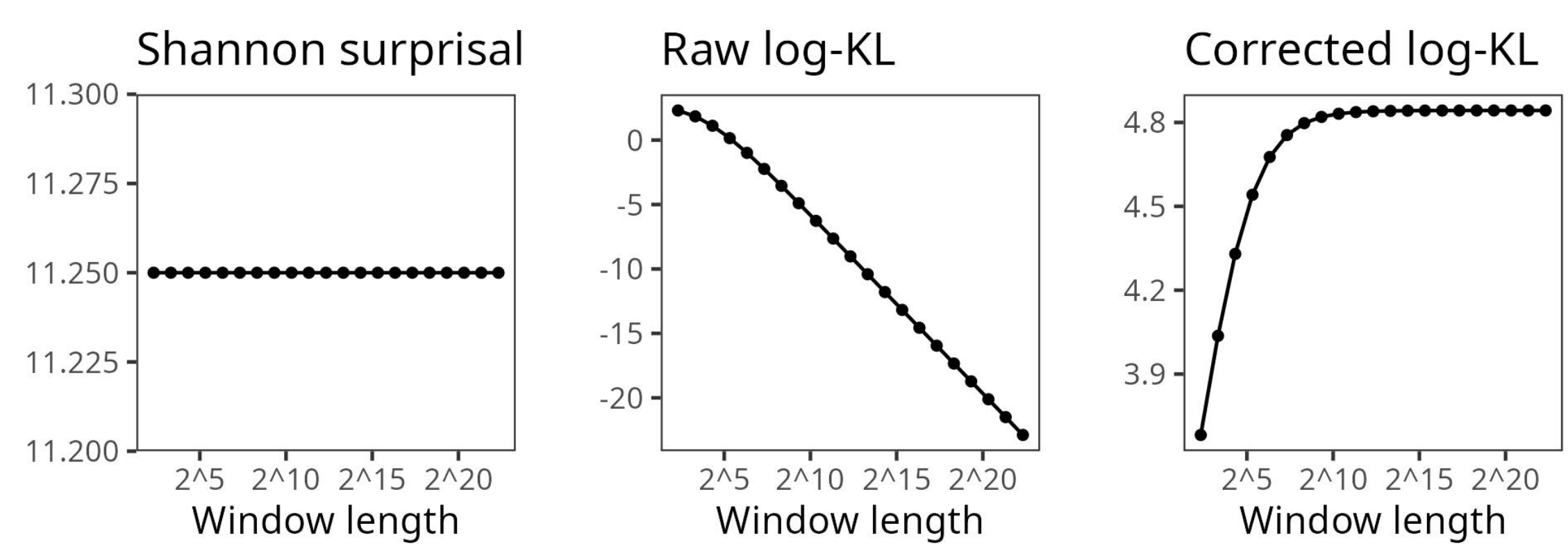

Fig. S6 Shannon surprisal of a new observation does not depend on the number of points sampled from a constant data-generating process (window length *w*), but KL divergence drops approximately linearly with *w*. This dependence of KL on *w* can be canceled by adding 2log(w), which becomes increasingly precise as *w* increases. Data: {0, 0, 0, -1, 1} repeated *w*/5 times and followed by the test value of {3}. Code in supplements, file fig_S6_window-dependence.R.
